# Amyotrophic lateral sclerosis-motor neuron disease, monoclonal gammopathy, hyperparathyroidism, and B12 deficiency: case report and review of the literature

**DOI:** 10.1186/1752-1947-4-298

**Published:** 2010-09-01

**Authors:** Richard A Rison, Said R Beydoun

**Affiliations:** 1 Neurology Consultants Medical Group, Presbyterian Intercommunity Hospital, Whittier, CA, USA; 2 Department of Neurology, University of Southern California, Keck School of Medicine, Los Angeles County Medical Center, Los Angeles, CA, USA

## Abstract

**Introduction:**

Amyotrophic lateral sclerosis (the most common form of motor neuron disease) is a progressive and devastating disease involving both lower and upper motor neurons, typically following a relentless path towards death. Given the gravity of this diagnosis, all efforts must be made by the clinician to exclude alternative and more treatable entities. Frequent serology testing involves searching for treatable disorders, including vitamin B12 deficiency, parathyroid anomalies, and monoclonal gammopathies.

**Case presentation:**

We present the case of a 78-year-old Caucasian man with all three of the aforementioned commonly searched for disorders during an investigation for amyotrophic lateral sclerosis.

**Conclusions:**

The clinical utility of these common tests and what they ultimately mean in patients with amyotrophic lateral sclerosis is discussed, along with a review of the literature.

## Introduction

Amyotrophic lateral sclerosis (ALS) is a progressive and devastating neurodegenerative disorder that affects primarily motor neurons, and is the most common type of motor neuron disorder. Because of the near-uniform 'kiss of death' implications that a diagnosis of ALS carries, all efforts must be made to exclude alternative diagnoses. Typical investigations look for any potential treatable cause of the patient's condition. In addition to electrodiagnostic studies, the usual investigations include neuroimaging studies to exclude anatomic structural processes such as cervical myelopathies, and typical laboratory investigations to search for any potential treatable metabolic abnormality. In particular, among the most common laboratory tests used are those for vitamin B12 levels (to rule out subacute combined degeneration), parathyroid hormone levels (to rule out hyperparathyroidism), and serum protein electrophoresis with immunofixation (to rule out multiple myeloma or monoclonal gammopathy of undetermined significance (MGUS)).

## Case presentation

A 78-year-old Caucasian man presented to our hospital with a history of weakness in the left arm and shoulder, with discomfort and difficulty dressing himself, for the past one and a half months. Initially he had attributed his issues to a prior rotator cuff injury. He then noted progressive shrinking in the muscles of his left arm and hand with decreased grip strength (overall he felt that he lost about 80% strength in his left arm) and developed uncomfortable 'charley horses' (painful spasms or cramps) in his left leg. There were no sensory, swallowing, or visual issues, and he denied having experienced any head or neck trauma. His medical history was significant for hypertension, coronary artery disease, hypercholesterolemia, hypothyroidism, and peptic ulcer disease.

Results of a neurological examination showed atrophy in the left biceps and deltoid and also the left first dorsal interossei (DI) muscle. Fasiculations were noted in the left forearm and left first dorsal interossei. Strength testing results, using Medical Research Council (MRC) grades, were as follows: left biceps 4/5, deltoid 4/5, and diminished grip strength 4/5. Strength testing in the left abductor digiti minimi (ADM) was 5-/5, left flexor digitorum profundus (FDP) 1 and 2 was 5-/5, left extensor carpi radialis longus (ECRL) 4+/5, and 4/5 in the left infraspinatous. Diminished grip strength (4/5) of the left hand was also noted. Normal strength testing (5/5) was noted in the right arm and bilateral lower extremities. Deep tendon reflexes were 1/4 in the left brachioradialis, biceps, and triceps and 2/4 for others. His toes were downgoing bilaterally. A mild gait imbalance was observed. His sensory examination was completely intact. No coordination deficits were seen. No cranial nerve deficits were observed except for mild tongue fasciculations. His speech was fluent without dysarthria or dysphasia.

An electrodiagnostic study performed the same week showed low amplitudes in the left upper extremity motor nerve compound muscle action potentials with intact sensory nerve action potential responses. There was no evidence of any abnormal temporal dispersion or conduction block in multiple nerves tested. There were 1-2+ fibrillation potentials and positive sharp waves in the left deltoid, triceps, biceps, flexor carpi radialis (FCR), first DI, tibialis anterior, and bilateral gastrocnemius medial heads. His tongue showed a discrete firing pattern without abnormal resting activity.

The results of neuroimaging studies of the spine revealed age-related degenerative joint and disc disease with spondylosis, but nothing that was felt would account for his clinical condition. The working diagnosis at this point was motor neuron disease (MND) probably secondary to ALS (or 'clinically possible ALS', via El Escorial criteria).

Further laboratory investigations revealed a monoclonal gammopathy (IgGλ subtype) (2,019 mg/dL, with the normal range being 694 to 1618 mg/dL) (κ to λ ratio of 1:2, with the normal ratio being 2:1) via both serum protein electrophoresis and serum immunofixation with leukopenia and anemia (moderate normochromic normocytic) along with B12 deficiency (116 pg/ml). Homocysteine was elevated (43.2 μmol/L, with the normal level being <11.4 μmol/L). Parietal antibody test results were positive (1:80, with normal results being <1:20) and an intrinsic factor antibody test result was positive, and for these reasons it was felt that our patient was B12 deficient. His erythrocyte sedimentation rate (ESR) was slightly elevated at 40 mm/hour (the normal range being within 0 to 20 mm/hour) but an anti-nuclear antibody screen test result was negative. It was therefore felt that the elevated ESR was due to anemia and/or monoclonal gammopathy rather than an underlying autoimmune process. Motor and sensory neuropathy panels and paraneoplastic panel test results were negative.

In order to exclude any potentially treatable causes of MND, our patient underwent a bone marrow biopsy to exclude any plasma cell dyscrasia. The results showed hypocellular marrow with a hematopoietic cellularity of 20-40% (overall 30%) with no evidence of any granuloma or lymphoma, or tumor. Immunophenotyping data did not show any evidence of neoplasia. There was no evidence of any myeloproliferative disorder. Our patient also underwent a colonoscopy, which showed multiple polyps but no evidence of any tumor. Our patient was given B12 supplements via injection by a hematologist, with a diagnosis of monoclonal gammopathy of undetermined significance (MGUS), B12 deficiency, and pernicious anemia.

Our patient's symptoms progressed to weakness in the left shoulder with increasing weakness in the left arm, and he underwent subsequent re-evaluation at a local university neuromuscular department approximately 2 months later. The re-examination showed atrophy in the bilateral spinati (left > right) along with persistent atrophy in the left first DI, left thenar, and left deltoid and biceps. Strength testing showed deltoid less than antigravity with 4-/5 biceps, 4-/5 triceps, and wrist extensors less than gravity on the left side with an inability to extend the fingers. Wrist flexion and finger flexion results were 4+/5 and interossei 3/5. In the right upper extremity the thenar group was 4-/5, infraspinatous 4/5, supraspinatous 4/5, deltoid 5-/5, biceps 5-/5, and the pectoralis 4-/5. The forearm muscles and hand muscles on the right side were in the 4+ to 5- range. Fasiculations were noted in the upper extremity muscles in a scattered distribution, predominately proximally.

A repeat electrodiagnostic study preformed approximately three to four weeks later revealed active denervation in multiple myotomes in both the upper and lower extremities with chronic denervation. Motor axonal loss changes were noted without conduction block.

Further laboratory test results that week included the following: parathyroid hormone (PTH) was elevated (155 pg/mL, normal range 10 to 65 pg/mL) and the calcium level was normal (9.6 mg/dL, normal range 8.8 to 10.3 mg/dL). Our patient's renal function was normal. Unfortunately, 25-hydroxy vitamin D levels were not tested.

The university diagnosis at that time was 'motor neuron disease confounded by monoclonal gammopathy and possible hyperparathyroidism'. This corresponded with the El Escorial criteria classification of 'clinically probable laboratory supported ALS'. A trial of intravenous immunoglobulin was considered for our patient, but not recommended pending further evaluation.

Our patient was seen by an endocrinologist who made a diagnosis of possible hyperparathyroidism (normocalcemic hyperparathyroidism), and an ultrasound study of the thyroid and parathyroid glands was performed one month later, showing a coarsened texture of the thyroid parenchyma consistent with diffuse pathology. However, no focal mass was seen. An enlarged parathyroid gland was not detected.

In light of the dire prognosis that MND carries, and the plausibility of hyperparathyroidism causing the MND, consultation with a vascular surgeon was arranged for consideration of a parathyroidectomy. At last known follow-up our patient had continued to deteriorate.

## Discussion

ALS is a progressive and devastating neurodegenerative disorder that affects primarily motor neurons. It is the most common type of motor neuron disorder. The characteristic form of this disease features the simultaneous presence of both upper motor neuron (UMN) and lower motor neuron (LMN) signs, with progression from one region of the neuraxis to the next. Many cases of ALS will begin with the LMN form and then with time progress to show UMN involvement. Most ALS is sporadic, and men tend to develop ALS more often than women with a male/female ratio of about 2:1. The incidence of the disease increases with age, with a peak occurrence between 55 and 75 years of age [1]. Treatment is symptomatic, with riluzole extending survival by 12% [2]. Death, usually from respiratory compromise, occurs approximately three years after onset of symptoms [1].

Because of the near-uniform 'kiss of death' implications that a diagnosis of ALS carries, all efforts must be sought to exclude alternative diagnoses. Typical investigations look for any potential treatable cause of the patient's condition. In addition to electrodiagnostic studies, investigations usually include neuroimaging studies to exclude anatomic structural processes such as cervical myelopathies, and typical laboratory investigations to search for any potential treatable metabolic abnormality. In particular, among the most common laboratory tests ordered are ones for vitamin B12 levels (to rule out subacute combined degeneration), parathyroid hormone levels (to rule out hyperparathyroidism), and serum protein electrophoresis with immunofixation (to rule out multiple myeloma or MGUS).

### Gammopathies and motor neuron disease

There have been various reports of patients with both monoclonal gammopathy and MND (see below). However, before a discussion of this it is useful to review the basic nomenclature, prevalence, and terminology. Lymphomas such as multiple myeloma and its precursor MGUS are in a different category to the myeloproliferative disorders/neoplasms (MPN). Myeloproliferative disorders/neoplasms include chronic myelogenous leukemia, polycythemia vera, essential thrombocythemia, primary myelofibrosis, chronic neutrophilic leukemia, chronic eosinophilic leukemia/hypereosinophilic syndrome and mast cell disease. Myeloproliferative disorders/neoplasms may be diagnosed by morphological aspects, cytogenetics and fluorescence *in situ *hybridization in blood and bone marrow. Serum protein electrophoresis with immunofixation is useful to rule out multiple myeloma or MGUS (though does not necessarily exclude MPN). Alexianu *et al*. [3] provide a further review; monoclonal antibodies are produced by expanded single B-cell clones and are variously known as monoclonal protein, M protein, M component, monoclonal gammopathy, or paraprotein. They are classified as IgM, IgG, IgA, IgE, or IgD according to the heavy chain class. Monoclonal gammopathy can be associated with non-malignant or malignant lymphoproliferative B-cell disorders. The non-malignant monoclonal gammopathies have been referred to as 'monoclonal gammopathies of undetermined significance', but the term 'non-malignant monoclonal gammopathy' is preferred. Non-malignant monoclonal gammopathy is differentiated from malignant monoclonal gammopathy by a lower level of serum M protein, low or undetectable level of M protein in the urine, absence of other signs of systemic disease such as lytic bone lesions, anemia, hypercalcemia, or renal insufficiency, and fewer than 10% plasma cells or absence of lymphoid aggregates in the bone marrow. Monoclonal antibodies are found in 10% of patients with peripheral neuropathy of otherwise unknown etiology [4]. Most IgM monoclonal gammopathies associated with neuropathy exhibit autoantibody reactivity to neural antigens, but such autoantibody activity has not been associated with IgG or IgA monoclonal gammopathies. The prevalence of monoclonal gammopathy in the adult population is approximately 1%. Among patients older than 70 years of age, the frequency of monoclonal gammopathy was found to be 3%. The distribution of heavy chain classes in patients with non-malignant monoclonal gammopathy is 73% to 86% IgG, 0% to 14% IgM, and 11% to 14% IgA [3].

The literature suggests that patients with MND may have a higher incidence of lymphoproliferative disorders (LPD). The association between MND and LPD could be coincidental, but LPD seems to be disproportionately frequent in patients with MND compared to the population in general [5]. Despite an initial report suggesting that major improvements occurred sometimes coincidentally with reductions in paraprotein levels using prednisone, cyclophosphamide, chlorambucil and plasma exchange treatments even in some patients who had the clinical appearance of ALS [6], most of the subsequent literature argues against this, with less than successful trials using various immunomodulatory agents and plasma exchange. Gordon *et al*. [7] studied 26 patients with both MND and LPD. Most of the patients with MND with LPD had Hodgkin's or non-Hodgkin's lymphoma, such as myeloma or macroglobulinemia. Among these patients, few had a beneficial neurological response to immunotherapy, and most died of the neurological disease. Other reports highlight the association of MND and the presence of a lymphoplasmocytoid infiltration of Waldenstrom's macroglobulinemia in particular, and the disappointing lack of neuromuscular improvement following treatment of the underlying hemopathy with plasmapheresis and immunosuppressive therapy [5]. When MND does occur in association with LPD, it appears to have both UMN and LMN involvement compatible with a diagnosis of ALS [8]. Unfortunately, overall there does not appear to be a clinical benefit with the aforementioned treatments.

A malignant monoclonal gammopathy is a feature of lymphoproliferative disease and not of myeloproliferative neoplasms. Non-malignant monoclonal gammopathies can be associated with liver diseases, inflammation, or chronic lymphatic leukemia. A significant proportion of patients with ALS/MND will have a non-malignant monoclonal gammopathy. In fact, serum protein electrophoresis with immunofixation shows evidence of monoclonal immunoglobulin M (IgM) gammopathy in approximately 10% of patients with MND, the M proteins having specific activity against neuronal antigens. Various reports have also shown associations between MND and other paraproteins, including IgG, IgA, M proteins, Bence-Jones proteins, and polyclonal gammopathies. Rowland *et al*. [9] reported a patient with an IgMκM protein and ALS and reviewed the published cases of 14 other patients with MND and monoclonal gammopathy. In a literature review, Latov [10] found 19 cases of MND and monoclonal gammopathy. Patten [6] described four patients with ALS and IgG monoclonal gammopathy. Shy *et al*. [11] found that 10 of 206 patients (4.8%) with MND had M proteins. Of these patients, four had IgM and six had IgG. Of 100 control patients with other neurological diseases, only a single patient had an M protein. Subsequently, six patients with MND and M proteins were found, as well as three patients with polyclonal IgM elevation and two with Bence-Jones proteins. In 1987 Rudnicki *et al*. [12] studied two patients with MND and paraproteinemia. One had ALS and IgGλ monoclonal gammopathy. The second had slowly progressive muscular atrophy and an IgMκ paraprotein, followed by a biclonal gammopathy when an IgAκ paraprotein appeared. Treatment with immunosuppressive agents and plasmapheresis lowered the paraprotein serum concentration. The ALS syndrome progressed despite therapy. The other patient improved, was stable for several years, but then deteriorated despite continued therapy. Merlini *et al*. [13] reported on three patients with ALS, two of whom had an IgG monoclonal protein and one with biclonal gammopathy (IgGκ/IgAλ). Saito *et al*. [14] reviewed the presence of monoclonal immunoglobulin in the serum of multiple patients with MND with the incidence of paraproteins being 11.3%. The monoclonal components found were IgG (33%), IgM (33%) and IgA (33%). In six cases, four showed typical changes of ALS and the other two patients had pathological findings of spinal progressive muscular atrophy (SPMA) at autopsy. No malignancy was detected in any case. These results corroborate the concept of a probable association between MND and benign monoclonal gammopathy (plasma cell dyscrasias). Lavrnić *et al*. [15] found the prevalence of monoclonal gammopathy among patients with MND to be 6 out of 56 (10.7%). Of these six patients, four had an IgG and two had an IgA paraprotein. The clinical syndromes consisted of ALS in two patients, lower motor neuron syndrome with preserved reflexes in at least one limb in three patients, and motor neuropathy with multifocal conduction block in one patient. The presence of gammopathy appears to correlate with the absence of marked upper motor neuron involvement and with elevated cerebrospinal fluid (CSF) protein concentration. An underlying malignant disorder was ruled out in all six patients, and they were considered to have monoclonal gammopathy of undetermined significance (MGUS). Thus, there does not appear to be any clinical benefit of plasmapharesis or immunosuppressive treatments in these patients.

Although the occurrence of monoclonal gammopathy and motor neuron disease has been reported, the evidence of a causal relationship is limited.

### Hyperparathyroidism and motor neuron disease

The association of muscle weakness with primary hyperparathyroidism (PHP) dates back to the 1800s [16], and since then various patients have been reported with PHP, muscle weakness, hyper-reflexia, and muscle atrophy. There were even reports in the 1980s of patients with PHP, muscle weakness, hyper-reflexia with dysarthria and fasiculations who underwent parathyroid ademona resection and demonstrated improved muscle performance [17], and other case reports have suggested improvement in symptoms following treatment of PHP [18]. However, Rodriguez *et al*. [19] reported on a series of patients diagnosed with ALS and concluded that there was no pathogenic association between thyroid dysfunction or alteration of phosphate calcium metabolism and ALS. Perhaps most convincing is the study by Jackson *et al*. [20] who reported on five patients with ALS and PHP that underwent parathyroid ademona resection. Each patient had subsequent normalization of serum calcium and PTH levels, but unfortunately they all had progressive weakness eventually resulting in death within 3 years following parathyroidectomy.

There have been some interesting associations reported with ALS/MND, calcium and vitamin D metabolism. In the 1970s Patten and Mallette [21] published a retrospective study of associated abnormalities in MND and found that over 50% of patients had radiographic abnormalities of bone and over 20% had serum calcium concentrations out of the range observed in normal controls. The authors suggested that disturbances in calcium metabolism may stimulate MND and place patients with both PHP and secondary hyperparathyroidism (SHP) at risk for ALS. Exactly how this occurs is far from clear. The causative role of trace elements in the pathogenesis of ALS has been studied. In animal studies it has been postulated that chronic environmental deficiencies of calcium and magnesium may provoke secondary hyperparathyroidism, resulting in increased intestinal absorption of toxic metals. This leads to the presence of excess levels of divalent or trivalent cations, which in turn leads to the mobilization of calcium and metals from the bone and deposition of these elements in the nervous tissue ('metal-induced calcifying generation of the CNS') [22]. In human reports, however, confirmation of this has been lacking. In a study of patients with Guamanian neurodegenerative disease and Chamorro control subjects using blood serum, urine, nail, and hair heavy metal concentrations, Ahlskog *et al*. [22] were unable to find any evidence of abnormalities of calcium metabolism or heavy metal absorption as a major causative factor in the development of neurodegenerative disease on the island of Guam.

Interestingly, patients with ALS have been found to have abnormalities in Vitamin D levels. Sato *et al*. [23] found reduced serum concentrations of 25-hydroxyvitamin D (25-OHD) in patients with ALS than in controls along elevated PTH levels and ionized calcium. Whitaker *et al*. [24] reported a patient thought to have lower MND and found to have vitamin D deficiency and secondary hyperparathyroidism who showed substantial clinical improvement following vitamin D therapy.

There are similarities in the neuromuscular symptoms and signs with PHP and ALS. Patients with PHP may develop muscle weakness and atrophy involving the lower extremities but the weakness tends to be symmetric and involves the proximal muscles predominately. Patients with PHP often have brisk muscle stretch reflexes with plantar responses (although there have been reports of patients with extensor plantar responses). Muscle cramps have been reported in around 50% of patients with PHP. Severe respiratory muscle involvement has been reported in PHP, and bulbar involvement resulting in hoarseness and dysphasia, as well as abnormal tongue movements, have also been seen [25]. There are several important differences in symptoms between ALS and PHP, however. Patients with PHP often have stocking-glove loss of pain and vibratory sensation as well as parathesias [25]. Patients with PHP may also have associated ataxia, decreased arm swing, abnormal hand and arm posturing [25] along with poor memory, slow mentation, disorientation, emotional lability, personality changes, anxiety, and hallucinations (as discussed in Jackson *et al*. [20]).

The mechanism of weakness in PHP is unknown. PTH enhances muscle proteolysis and impairs energy production, transfer, and utilization. PTH may also diminish the sensitivity of contractile myofibrillary proteins to calcium and activate a cytoplasmic protease, thus impairing muscle bioenergetics [26]. Calcium and phosphorous levels do no correlate well with the degree of neuromuscular symptoms [25,27]. Muscle biopsies usually demonstrate non-specific features including atrophy (predominately type 2 fibers) along with occasional group atrophy and fiber-type grouping [25]. Electromyogram (EMG) results can be normal or show small polyphasic motor unit potentials with early recruitment suggestive of myopathy [25,27]. There have been reports, however, of neurogenic features on EMG including fibrillations, fasiculations, large polyphasic motor units, and decreased recruitment although this is rare [25] (as discussed in Jackson *et al*. [20]).

Although there are interesting correlations between PHP and SHP, MND/ALS with aberrant calcium, vitamin D, and PTH metabolism, most of the literature does not indicate a conclusive relationship between ALS and PHP/SHP and treatment of PHP/SHP does not lead to improvement of MND.

### Vitamin B12 levels and MND/ALS

B12 deficiency is associated with megaloblastic anemia, glossitis, dementia, peripheral neuropathy and myelopathy. In particular, deficiencies of vitamin B12 may cause subacute combined degeneration of the spinal cord. This disorder shares upper motor neuron signs with ALS, and hence it is customary to measure B12 levels in the investigation of not only ALS but also all peripheral neuropathies because of the readily available treatments for deficiency.

Fortunately, B12-deficient neuropathies have different characteristics from that of MND/ALS. Although the clinical features of vitamin B12 deficiency may consist of a classic triad of weakness, sore tongue, and paresthesias, these are not usually the chief symptoms. Onset is often with a sensation of cold, numbness, or tightness in the tips of the toes and then in the fingertips, rarely with lancinating pains. Simultaneous involvement of arms and legs is uncommon, and onset in the arms is even more rare. Paresthesias are ascending and occasionally involve the trunk, leading to a sensation of constriction in the abdomen and chest. Patients who are not treated may develop limb weakness and ataxia (as discussed in Singh *et al*. [28]).

In 1991, Healton *et al*. [29] performed detailed neurological evaluations of patients who were vitamin B12 deficient. A total of 74% presented with neurological symptoms. Isolated numbness or paresthesias were present in 33%, gait abnormalities occurred in 12%, psychiatric or cognitive symptoms were noted in 3%, and visual symptoms were reported in 0.5%. Isolated neuropathy was reported in 25% of patients. Myelopathy occurred in 12% of cases. A combination of neuropathy and myelopathy was noted in 41%. Half of the patients had absent ankle reflexes with relative hyper-reflexia at the knees on presentation. Plantars were initially flexor and later extensor. A Hoffman sign was found in some cases. As the disease progressed, ascending loss of pinprick, light touch, and temperature sensation occurred. Later, depending on the predominance of posterior column versus cortical spinal tract involvement, ataxia or spastic paraplegia predominated followed by distal limb atrophy. Symptoms also included subacute progressive decrease in visual acuity, usually caused by bilateral optic neuropathy and rarely pseudotumor cerebri or optic neuritis. Rare autonomic features included orthostasis, sexual dysfunction, and bowel and bladder incontinence. Other symptoms included lightheadedness and impaired taste and smell. Non-neurological symptoms, some of which may also reflect autonomic nervous system involvement, were present in 26%. Constitutional symptoms, including anorexia and weight loss occurred in half. Low-grade fever that resolves with treatment occurred in 33% of cases. Other symptoms include fatigue and malaise. Cardiovascular symptoms include syncope, dyspnea, orthopnea, palpitations, and angina. Gastrointestinal symptoms include heartburn, flatulence, constipation, diarrhea, sore tongue, and early satiety (as discussed in Singh *et al*. [28]).

In the USA the prevalence of vitamin B12 deficiency is difficult to ascertain because of diverse etiologies and different assays (radioassay or chemiluminescence). Affected individuals may number 300,000 to 3,000,000 in the USA. Using the radioassay and a value less than 200 pg/mL, the prevalence of vitamin B12 deficiency is 3% to 16%. In a geriatric population using a radioassay cut-off of 300 pg/mL and elevated homocysteine (HC) and methylmalonic acid (MMA) levels, a prevalence of 21% was reported. In Europe, the prevalence of vitamin B12 deficiency is 1.6% to 10%. Pernicious anemia (PA) prevalence may be higher in white people and lower in Hispanic and black people. No known relationship exists between neurological symptoms and race. Studies in Africa and the USA have shown higher vitamin B12 and transcobalamin II levels in black than in white individuals. Additionally, blacks have lower HC levels and metabolize it more efficiently than whites. In Europe and Africa, the prevalence of PA is higher in older women than men (1.5:1), while in the USA no differences exist. Men have higher HC levels at all ages. PA occurs in people of all ages, but it is more common in people older than 40 to 70 years and, in particular, in people older than 65 years. In white people, the mean age of onset is 60; in black people, the mean age is 50 years (as discussed in Singh *et al*. [28]).

Treatment of ALS with vitamin B12 has been attempted without success [30]. It therefore appears that the simultaneous occurrence of NMD/ALS is by chance alone. Vitamin B12 therapy in ALS/MND is unsuccessful.

## Conclusions

In summary, we present an interesting case of a patient with ALS with parallel diagnoses of MGUS, possible hyperparathyroidism (normocalcemic), plus B12 deficiency. After review of the available literature, it was felt that these were chance occurrences and that treatment of these secondary entities does not affect the course of progressive ALS/MND. Symptoms of each may mimic ALS. Clinically, the presence of gammopathy appears to correlate with the absence of UMN involvement, slowly progressive muscular atrophy, and with elevated CSF protein. With PHP, clinical weakness tends to be symmetric, involves proximal muscles predominately, brisk muscle stretch reflexes are seen along with plantar responses and muscle cramps. Importantly, patients with PHP often also display non-motor findings such as ataxia, paresthesias, and cognitive slowing. Patients with vitamin B12 deficiency frequently develop paresthesias and dysesthesias with limb weakness and ataxia. Also, patients who are B12 deficient often have neuromyelopathic issues (subacute combined degeneration of the spinal cord) as well with cognitive symptoms. As neurologists we will still continue to search for alternative diagnoses in patients with suspected ALS, bearing in mind however that the clinical utility of these results may not affect the final outcome.

## Consent

Written informed consent for publication could not be obtained despite all reasonable attempts. Every effort has been made to protect the identity of our patient and there is no reason to believe that our patient would object to publication.

## Competing interests

The authors declare that they have no competing interests.

## Authors' contributions

RAR examined our patient, performed the electrodiagnostic studies, and wrote the manuscript. SRB examined our patient, performed repeat electrodiagnostic studies, and critically reviewed the manuscript offering suggestions and revisions. Both RAR and SRB are responsible for the intellectual content of the manuscript. Both authors approved the final manuscript.
